# Assessment of Personal Relaxation in Indoor-Air Environments: Study in Real Full-Scale Laboratory Houses

**DOI:** 10.3390/ijerph181910246

**Published:** 2021-09-29

**Authors:** Yoshitake Nakayama, Norimichi Suzuki, Hiroko Nakaoka, Kayo Tsumura, Kohki Takaguchi, Kazunari Takaya, Masamichi Hanazato, Emiko Todaka, Chisato Mori

**Affiliations:** 1 Center for Preventive Medical Sciences, Chiba University, 6-2-1 Kashiwanoha, Kashiwa, Chiba 277-0882, Japan; suzu-nori@chiba-u.jp (N.S.); hnakaoka@faculty.chiba-u.jp (H.N.); tsumu-kayo@chiba-u.jp (K.T.); k.takaguchi@chiba-u.jp (K.T.); hanazato@chiba-u.jp (M.H.); todakae@faculty.chiba-u.jp (E.T.); cmori@faculty.chiba-u.jp (C.M.); 2 Department of Bioenvironmental Medicine, Graduate School of Medicine, Chiba University, 1-8-1 Inohana, Chuo-ku, Chiba 260-8670, Japan; 3 Graduate School of Medical and Pharmaceutical Sciences, Chiba University, 1-8-1 Inohana, Chuo-ku, Chiba 260-8670, Japan; 4 National Institute of Occupational Safety and Health, 6-21-1 Nagao, Tama-ku, Kawasaki 214-8585, Japan; takaya-k@h.jniosh.johas.go.jp

**Keywords:** indoor-air quality, volatile organic compounds, odor, questionnaire, electroencephalogram, laboratory houses

## Abstract

The relationship between chemical concentrations in indoor air and the human sense of comfort and relaxation have been reported. We investigated the effect of the sum of volatile organic compounds (ΣVOCs; sum of 79 VOCs) on the level of relaxation in two laboratory houses with almost identical interior and exterior appearances. The electroencephalogram (EEG) was monitored to evaluate the degree of personal relaxation objectively. The experiments were conducted in laboratory houses (LH) A and B with lower and higher levels of ΣVOCs, respectively. A total of 168 healthy volunteers participated, who each performed the task for 20 min, followed by a 10-min break, and EEG was measured during the break. Simultaneously as subjective evaluations, the participants were asked to fill a questionnaire regarding the intensity of odor and preference for the air quality in each LH. The subjective evaluation showed a significant association between ΣVOCs and participants’ relaxation (OR: 2.86, 95%CI: 1.24–6.61), and the objective evaluation indicated that the participants were more relaxed in the LH with lower levels of ΣVOCs than that with higher levels (OR: 3.03, 95%CI: 1.23–7.50). Therefore, the reduction of ΣVOCs and odors in indoor air would have an effect, which is the promotion of relaxation.

## 1. Introduction

Improving the air quality of indoor spaces can prevent diseases and symptoms [1,2]. For example, reducing the concentration of sum of volatile organic compounds (ΣVOCs) in living environments and the odor of specific chemicals can reduce the occurrence of building related symptoms (BRSs) [3,4]. In epidemiological studies, lifestyle changes such as frequent ventilation and cleaning were shown to reduce the occurrence of BRSs, asthma, and other allergic symptoms [5,6,7,8,9]. Very recently, improving the indoor-air environment through ventilation has proven effective against COVID19 infection [10,11].

Other investigators have studied the physiological, psychological, and sociological impacts of improving indoor environments. For instance, the indoor environment is known to affect daily life and production efficiency. In a large cross-sectional study of 7441 employees occupying 167 office buildings in eight European countries, the most important environmental factor associated with indoor comfort and indoor environmental quality was noise, followed by air quality, light, and heat [12]. The estimated annual productivity gain from improving the air environment was 330 euros per employee. Despite their obvious importance, the potential health and productivity benefits appear not to be integrated into the traditional economic calculations associated with building design and operation [13]. In evaluations of indoor-air quality (IAQ), odor removal through ventilation and filtering was found to improve the performance and subjective responses of workers [14]. The potential benefits of odor mitigation were also highlighted by Fisk et al. [15]. Meanwhile, indoor thermal comfort mitigates BRSs [16,17], as evidenced by the improved relaxation, recovery rate, and work efficiency in comfortable thermal environments evaluated by electroencephalogram (EEG) and facial expression analysis. Carrer et al. revealed that non-smoking programs, stress-free environments, and IAQ management in the work environment improved the health of workers [18]. They clarified the relationships between a wide variety of indoor environmental factors and the activity, quality, and health of employees.

However, whether reducing the ΣVOCs in living environments improves the comfort and relaxation of inhabitants has not been investigated in detail. In Japan, the Ministry of Health, Labor and Welfare formulated the “Second Phase of the National Health Promotion Movement for the 21st Century (Health Nippon 21 (Phase 2))” in 2012 [19]. This plan aims to realize a healthy, mindful, and vibrant society through all life stages. According to the 2018 interim report, 80% of the improvements have been achieved in the social environment, but improving individual lifestyles (especially rest) was reported to be a future challenge. A unit space composed of wood appears to promote psychological relaxation through visual impacts [20], and certain fragrances enhance concentration and performance [21]. However, adjusting for other environmental factors and accounting for individual attributes, preconceptions, and preferences remain problematic.

The Center for Preventive Medical Sciences of Chiba University has launched the “Chemiless Town Project Phase 3,” which evaluates human sensory perception while measuring chemical substance levels on the same day. The project hypothesizes that improving the air environment could prevent BRSs and promote human health. The current study investigates the relationship between VOC levels in indoor-air environments and the recovery rates from physical and mental fatigue. To validate the data obtained from the relationship between VOC levels in indoor-air environments and the recovery rates from physical and mental fatigue, psychological (subjective) and biological (objective) measurements such as sensation and emotion in a laboratory house (LH) stay evaluation test were combined. The data on preferences, air-quality ratings, and feelings of comfort and relaxation were quantitatively analyzed.

## 2. Materials and Methods

### 2.1. Experimental Design and Test Location

**Figure 1:**
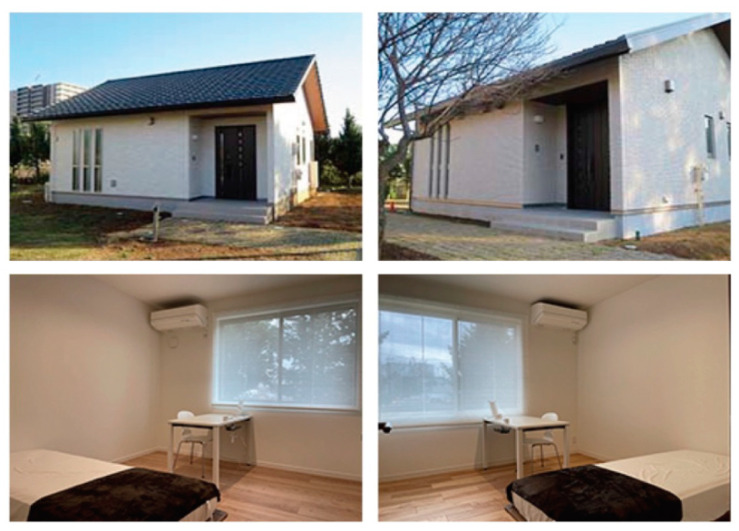
Evaluation tests were conducted in the LHs. Upper left: exterior of LH-A (typical Japanese wooden house), bottom left: bedroom of LH-A, upper right: exterior of LH-B (light-gage steel-structured house), bottom right: bedroom of LH-B.

The experiment was carried out from May to October 2018. Evaluation tests were conducted on 168 healthy volunteers stayed in two LH living rooms with almost identical interior and exterior appearances, furnishings, room temperature, relative humidity, noise, and illumination (Figure 1). There were no significant differences between LHs in environmental parameters except ΣVOCs [3]. In addition, the experiments were conducted on the same date and time at each LH. Since they were built next to each other, the outside air conditions were almost the same. However, the structure materials of the two LHs are different. The structure of LH-A is a typical Japanese wooden house constructed from timber and the average ΣVOCs was 3629 μg/m^3^, sum of 79 quantified substances. In contrast, LH-B is an air-quality conscious house with a light-gage steel structure and the average ΣVOCs was 55 μg/m^3^ [3]. Before the evaluation, the test procedures were informed and written consents were obtained from all the participants.

Prior to the study, participants were requested to refrain from applying perfumes, antiperspirants, and other scented substances. The study adopted the blind model. Participants were informed only that the experiment was a study on air quality, not on the air-quality status of their allocated LH. The study protocol was approved by the Research Ethics Committee of the Graduate School of Medicine, Chiba University (Approval No. 2737). All study procedures were conducted according to the principles of World Medical Association Declaration of Helsinki.

### 2.2. Indoor-Air Sampling and Analysis

The indoor air samples were collected in the morning of each evaluation test. The details of chemical analysis and environmental data were presented in our previous report on BRSs prevention [3]. The list of measured VOCs in this experiment can be found at the following URL: https://doi.org/10.1016/j.scitotenv.2020.141635 (accessed on 25 August 2021).

### 2.3. Evaluation Test

**Figure 2:**
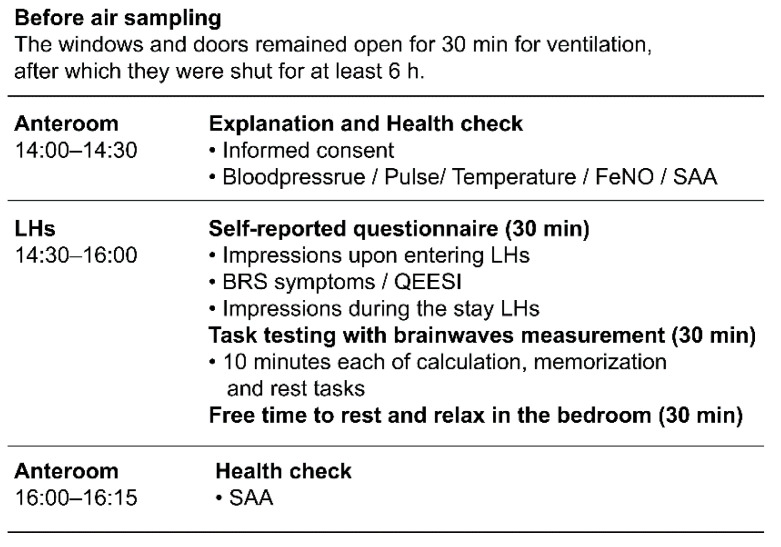
Work flowchart of the evaluation test.

Experiments were conducted once a day in each LH to avoid the influence among participants, such as odor or VOCs emitted from individuals. As Figure 2 shows, VOCs in indoor air were sampled in the morning, and in the afternoon, evaluation tests were conducted in each of LH. At the time of participation, the subjects’ blood pressure, body temperature, and exhaled nitric oxide concentration (FeNO) were measured as a health check, and salivary amylase assay (SAA) was determined to see their stress state. During the stay, the participants completed a 30-min questionnaire on their personal attributes and the environment (including air quality) in which they were staying. Then their brainwaves were measured during the three tasks that were memory, calculation, and resting. For the remaining time participants relaxed in the room (Figure 2). The questionnaire on personal attributes included the BRS-related items used in our previous study: age, gender, sensitivity to chemicals in Quick Environmental Exposure and Sensitivity Inventory (QEESI), presence of allergic diseases, smoking status, and occurrence of BRSs [3]. The questionnaire on the staying environment evaluated the participants’ impressions of the room brightness, heat, humidity, size, noise, openness, and naturalness on a 5-point bipolar scale.

After completing the evaluation tests, the health conditions of each participant were checked. The characteristics of the participants of the experiment were shown elsewhere [3]. Table 1 shows the number and percentage of participants’ results for SAA and FeNO concentrations. SAA values in the 0–30 KU/L range were considered normal for adults, and values above 31 KU/L were considered high. Meanwhile, FeNO levels below and above 36.8 ppb were considered as normal and high, respectively [22]. The SAA was measured using a salivary amylase monitor (DM-3.1/Nipro Corporation, Osaka, Japan), and the FeNO was measured using a portable nitric oxide monitor (Niox Vero/Circassia AB, Uppsala, Sweden).

### 2.4. Questionnaire Survey on Air Quality

The survey on air quality consisted of two questionnaires, one evaluating the participants’ impressions on air quality upon entering the LHs, the other evaluating their impressions during their stay. When entering the room, the participants were asked to rate the odor in the room (strong smell, moderate smell, faint smell, or no smell), along with the following items: odor preference (extreme dislike, dislike, neither like nor dislike, like, or greatly like), overall air-quality satisfaction (dissatisfied, somewhat dissatisfied, neither satisfied nor dissatisfied, somewhat satisfied, and satisfied), and comfort and relaxation (dissatisfied, somewhat dissatisfied, neither satisfied nor dissatisfied, somewhat satisfied, and very satisfied) [16]. The participants responded in the online questionnaire Soft Questant (Macromill, Inc., Tokyo, Japan), following the instructions and questions displayed on the PC screen.

### 2.5. Task Test

**Figure 3:**
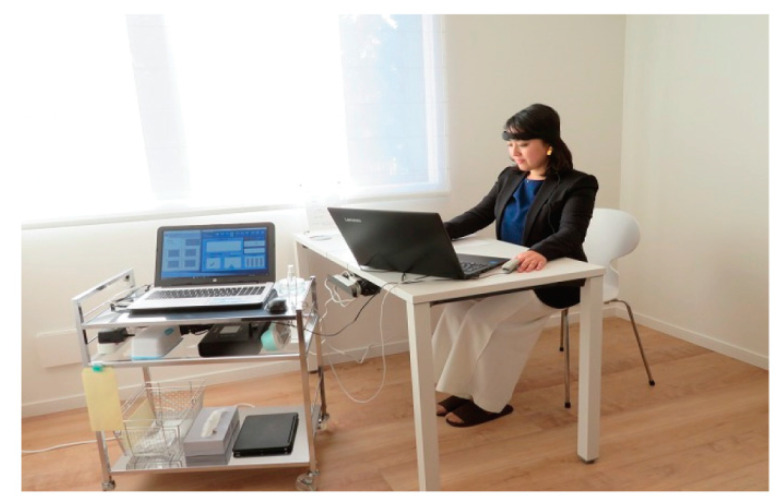
Subject attaches the probe to its head. After starting the EEG measurement, they begin to perform the tasks of calculation, memorization, and rest, according to the PC navigation.

During the LH stay, the electroencephalogram (EEG) fluctuations of the participants were measured during a 30-min task (Figure 3). The task included 10 min each of calculations, memorization [23], and then resting. The attendant left the room after explaining the installation of the EEG measurement device and task outline. The participants performed the task by themselves, following the video navigation with sound played from a PC. The EEG measurements were collected by a biofeedback device (Brain-ProFM-929, Futek Electronics, Kanagawa, Japan). A special probe was attached to the subject’s head (Figure 3). Following the International 10/20 method, the search, ground, and reference electrodes were selected as Fp2, which is a placement position, in the prefrontal cortex, Fp1, and A1 in the left ear, respectively. Measurements were continuously acquired for 30 min at a sampling rate of one per second. The participants were instructed to refrain from large body movements to prevent the head’s large movements and large blinks during the task, and to close their eyes and breathe slowly and deeply in a comfortable posture while resting [24,25].

### 2.6. Definition of EEG Frequency Bands

The EEG measurements were measured from 3.0 to 30.0 Hz (at 0.5-Hz intervals), covering the range of delta waves that dominate deep sleep (3.0–3.5 Hz), theta waves that tend to appear while dozing (4.0–6.0 Hz), alpha waves that occupy different ranges (6.5–8.5 Hz and 9.0–11.0 Hz during relaxation, and 9.0–11.0 Hz and 11.5–13.0 Hz during relaxation and concentration), and β waves that dominate concentration and working states (13.5–30.0 Hz). The strengths of the potentials were also measured. To remove artifacts associated with body movements, data below 3.5 Hz and above 20 μV were excluded from the analysis. When defining the bands, Pitchford et al. [25] assigned 8–13 Hz to the alpha band and 13–35 Hz to the beta band. They revealed a positive relationship between the resting alpha–beta EEG characteristics and attention to the task (after resting). Tenke CE et al. [26] compared the brainwave characteristics of rest and task performances. They similarly assigned 8–12 Hz as the alpha band. As mentioned above, the definitions of the α and β bands are different. In the present study, we focused on the resting time after the calculation and memorization tasks, and defined the alpha band as the most commonly observed frequency band during the relaxed state. The beta band was then defined as the frequency band found during arousal (concentration). Furthermore, the percentage of alpha/beta values that differed between the rest, and calculation and memory task periods was defined as the increase or decrease rate of the alpha/beta values.

## 3. Statistical Analysis

### 3.1. Subjective Evaluation

The subjective air-quality evaluations were stratified and tabulated into LH-A and LH-B. Variables related to odor were defined as “no smell” (no odor impact) and “smell” for others. Odor preference was categorized into “high” (like, greatly like) and “low/neutral.” The air-quality variable was defined as “high” (somewhat satisfied, very satisfied) and “low/neutral.” The significance of the evaluation differences among the LHs was examined by the Mann–Whitney U test.

### 3.2. Objective Evaluation

The frequency distribution of the percentage increase/decreases of α/β values of the brainwaves was analyzed and is presented in supplementary materials Figure S1. The Shapiro–Wilk test results indicated a non-normal distribution with *p* < 0.05. The results of the group with the highest quartile (feeling the strongest sense of relaxation) were compared with those of the other groups. Then, the association between the results of each of the subjective and objective methods was examined using Spearman’s rank correlation analysis.

### 3.3. Multivariate Analysis

Each subjective and objective evaluation was applied as the dependent variable in a binomial logistic regression analysis. The independent variables were age, gender, QEESI, physical condition, history of allergy, smoking history, and building of stay (LH), as in our previous report [3], and three additional variables: SAA result, FeNO level, and BRSs to adjust for the subjects’ stress status, respiratory status, and BRS development, respectively. Multicollinearity was checked by Spearman’s rank correlation analysis. The correlation coefficients of all variables were below 0.4 at the *p* < 0.05 significance level. In the multivariate analysis, the odds ratio (OR) and 95% confidence interval (95% CI) were calculated at the *p* < 0.05 significance level. All analyses were performed using SPSS statistics software (version 26.0 for Mac; SPSS Inc., Chicago, IL, USA).

## 4. Results

Table 2 shows the descriptive statistics of the subjective air-quality evaluation. The results are stratified into LH-A and LH-B. The proportion of participants “not bothered by the odor” when entering the room and throughout the stay ranged from 27.1% to 50% in LH-A and from 67.6% to 85.9% in LH-B, indicating that the participants were less affected by odors in LH-B than in LH-A. The difference in the “not bothered by odor” ratings between the two LH groups was statistically significant (*p* < 0.05). No significant difference between the “like” ratings of the odor was found between the two house groups (31.4% for LH-A and 33.8% for LH-B; *p* < 0.175). The air quality was rated as “high” by 54.3% of participants in the LH-A group and 73.2% by those in the LH-B group. This difference was statistically significant (*p* < 0.05). Meanwhile, 64.2% and 76.0% of the participants in the LH-A and LH-B groups, respectively, felt relaxed during their homestay (the higher rating in LH-B was significant with *p* < 0.05). After analyzing the association between the LHs, the subjective evaluations of the building environment other than air quality did not significantly differ between the two groups (Table 3).

In the objective evaluation, the alpha/beta rates of the participants’ brainwaves tended to increase more during the rest time than during the task time (84.3%, supplementary materials Figure S1), indicating that the participants were more relaxed while resting. The increase/decrease rates of the α/β values were significantly higher in the LH-B group than in the LH-A group (*p* < 0.05; supplementary materials Figure S2). The association between the results of the subjective and objective methods, it showed that there was a slight correlation, r: 0.196 and *p*-value < 0.05.

In each subjective and objective evaluation of relaxation, the results significantly differed between the LH-A and LH-B groups. Therefore, we conducted a binomial logistic regression analysis with each subjective and objective evaluation as the dependent variables, and the personal attributes and LHs as independent variables. The results are shown in Table 4 and Table 5. In the odor evaluation, the proportion of participants “not bothered by the odor” throughout the stay was significantly higher in LH-B than in LH-A. Among the participants who were no longer bothered by the odor during their stay at LHs, females were significantly more in number than males (3.17; 95% CI: 1.06–9.51). Also, those who were bothered by the odor were significantly more likely to complain of BRSs (0.06; 95% CI: 0.01–0.37). Favorableness of the odor was not associated with LH. The overall air quality was more highly rated in the LH-B group than in the LH-A group (OR: 2.57, 95% CI: 1.15–5.73), and the rating tended to decrease for those with history of allergy, high FeNO levels, and increased age (40 s and 50 s). Relaxation and comfort were rated significantly more highly in LH-B than in LH-A (OR: 2.86, 95% CI: 1.24–6.61), and the increase/decrease rates of the EEG alpha/beta values were also significantly higher in LH-B than in LH-A (OR: 3.03, 95% CI: 1.23–7.50). The furniture, such as beds, desks, and chairs in both LHs were the same, and indoor environmental factors such as room temperature, relative humidity, noise, and illumination were similarly controlled between the LHs. It was reported in our previous study that the difference of ΣVOCs between two LHs was significant even when the other environmental factors were analyzed as covariates [3]. Then the results indicate that LHs with different ΣVOCs significantly affect the subjective and objective evaluations, even after adjusting for individual attributes.

## 5. Discussion

Employing subjective and objective methods, we examined the factors that promote indoor comfort and relaxation. The results suggested that comfort and relaxation were more enhanced in LH-B (with fewer ΣVOCs) than in LH-A. Although LH-A received reasonably high ratings, and some level of positive evaluation was obtained in both LHs, LH-B was superior to LH-A. From a health-promotional viewpoint, odor exposure is a potential influencing factor of subjective comfort and relaxation. Previous studies have also reported the positive and negative effects of odor exposure on comfort, relaxation, and performance. In the present study, approximately half of the odor, air quality, and relaxation evaluations in LH-A were positive. This result probably reflects differences in personal sensitivity to odors and preference for certain odors.

Wolkoff and Novakova reported that emotional responses to odor (whether positive or negative) are discriminated, adjusted, and evaluated according to individual consciousness and past experiences [27,28]. In LH-A, we could infer the impacts of VOCs and odors peculiar to wooden buildings, which were identified in this house. Previous studies have reported that olfactory stimulation by VOCs, including δ-cadinene, 4-epicubebol, cubebol, and other sesquiterpenes derived from cedarwood, can exert a refreshing psychological effect after stressful work [29,30]. Particularly in Japan, there is a historical and cultural significance that wood promotes health. In fact, wood is visually and sensorially perceived as “warm” and full of “natural goodness.” Favorably perceived odors such as aromas reduce stress and facilitate memory tasks. Aromas have provided significant benefits in comparison tests with placebos [21,31]. Therefore, the woody odors in LH-A might have influenced the comfort and relaxation evaluations of participants in this house. However, negative reports of odor exposure suggest that fragrances can also degrade human health [32]. As the magnitude of the effect of odor exposure depends on individual sensitivity or odor hedonics, and on the duration of the exposure (acute or chronic), the personal attributes, exposure dose, and duration of a fragrance must be appropriately considered [27].

In this study, the evaluations of “not being bothered by the odor” were higher throughout the stay than upon entering the room. This can be explained by conscious habituation to the continuous odor exposure. However, participants who reported BRSs during the 90-min stay also gave lower odor evaluations. Even after adjustment to the continuous odor exposure, some sense of discomfort remained in the consciousness of these participants. Owing to individual differences such as odor preferences, clarifying the mechanism and verifying the related factors in this situation is a difficult task [33]. In the future, detailed investigations should to clarify the relationship between human assessment on IAQ and composition of each VOC. At this stage, we expect that reducing the concentration of ΣVOCs (as done in LH-B) and creating a more odorless environment will promote relaxation. Dalton et al. emphasized the importance of an odorless indoor environment and suggested mask-wearing when an odorless environment is not possible [34]. Although under the premise of lower ΣVOCs levels in indoor air, it seemed to be necessary to further verify of controlling odors to suit individuals’ physical conditions and preferences to promote personal relaxation.

In this study, in order to verify the effects of ΣVOCs levels on personal relaxation as physical reactions, we attempted to use EEG as an objective evaluation. Objective evaluations can be performed by measuring stress status with salivary amylase [29,30] or measuring the heart rate variability as a proxy of relaxation level [35]. In this study, we measured the brainwaves because EEG does not interfere with clothing and task work, allowing continuous measurements with minimal tension, burden, or discomfort to participants. The α/β values, which indicate a relaxed state, were higher during the resting period than during the task period.

The measurement sites, power spectrum, and other interpretations of EEG have been variously reported. Wooden furniture exerts adsorptive, antibacterial, visual, and physiological effects that improve memory and thinking ability [20], as confirmed in an alpha–beta power spectrum analysis [24,35,36]. Wood also promotes attention to additive tasks and enhances the resting state. Temperature changes also affect concentration, work performance, comfort, and relaxation, and thermal effects can increase or decrease the theta power of brainwaves [17,37]. In both cases, a decrease in low-frequency energy and increase in high-frequency energy indicates a sense of comfort and relaxation [38]. These findings were replicated in the present study. Comparing the results between the different LHs with different ΣVOCs and adjusting for personal attributes and other environmental factors, the rates of the alpha/beta values were significantly higher in LH-B than in LH-A. The objective physical responses suggested that reducing the ΣVOCs levels can increase comfort and relaxation in buildings.

## 6. Conclusions

In subjective and objective evaluations, this study revealed that reducing the ΣVOCs concentration and creating an odorless environment might promote the comfort and relaxation of diverse people with different attributes and preferences. Besides preventing BRSs and sensitivity to multiple chemicals, improving the IAQ should improve the life quality of healthy people. Particularly in recent years, the number of highly confidential and insulated houses has increased to improve energy efficiency, and thermal environments have gained importance. Therefore, continuous investigations on air quality are imperative. Provided with the results of this study, housing providers and users can develop, disseminate, and expand high-quality living environments that prevent possible adverse health effects and promote health.

## Figures and Tables

**Table 1:** Results of participants’ salivary amylase and FeNo concentrations stratified for LHs (*n* = 141).

		LH-A (*n* = 70)		LH-B (*n* = 71)	
		*n*	%	*n*	%
Salivary amylase					
	Normal	61	87.1	60	84.5
	High	9	12.9	11	15.5
FeNO concentrations					
	Normal	52	74.3	53	74.6
	High	18	25.7	18	25.4

**Table 2:** Indoor-air characteristics in the laboratory houses evaluated in the questionnaire study (*n* = 141).

		LH-A (*n* = 70)		LH-B (*n* = 71)		
		*n*	%	*n*	%	
Odor when entering the room						
	Smell	50	71.4	21	29.6	*
	no smell	19	27.1	48	67.6	*
	(missing)	1	1.4	2	2.8	
Odor in the staying room						
	Smell	35	50.0	9	12.7	*
	no smell	35	50.0	61	85.9	*
	(missing)			1	1.4	
Appeal of odor						
	low/neutral	47	67.1	45	63.4	n.s.
	High	22	31.4	24	33.8	n.s.
	(missing)	1	1.4	2	2.8	
Air quality						
	low/neutral	32	45.7	18	25.4	*
	High	38	54.3	52	73.2	*
	(missing)			1	1.4	
Relaxation (comfort)						
	low/neutral	25	35.6	16	22.5	*
	High	32	45.7	28	39.4	*
	very high	13	18.5	26	36.6	*
	(missing)			1	1.4	

* *p* < 0.05, n.s.: not significant.

**Table 3:** Subjective evaluation of environmental factors of staying room. Significance difference test for LH-A and LH-B *p* value.

Brightness	Heat	Humidity	Size	Noisiness	Openness	Nature
0.391	0.190	0.271	0.213	0.277	0.325	0.731

**Table 4:** Results of logistic regression on the associations between subjective evaluation and personal and environmental factor.

Factors		Subjective					
		Odor			Preference Odor	Air Quality	Relax (Comfort)
		Entering Room		Staying Room			
		Odds Ratio (95% CI)		Odds Ratio (95% CI)	Odds Ratio (95% CI)	Odds Ratio (95% CI)	Odds Ratio (95% CI)
Gender							
	male	Ref.					
	female		1.19 (0.49–2.90)	3.17 (1.06–9.51) *	0.96 (0.39–2.36)	1.70 (0.68–4.24)	0.83 (0.33–2.07)
Age							
	20–29	Ref.					
	30–39		1.12 (0.40–3.13)	0.43 (0.12–1.54)	0.45 (0.16–1.25)	0.62 (0.22–1.77)	0.86 (0.31–2.39)
	40–49		0.68 (0.21–2.21)	0.19 (0.05–0.72)	0.00 (0.00-.) **	0.30 (0.10–0.91) *	0.50 (0.14–1.80)
	≥50		0.63 (0.17–2.39)	0.40 (0.08–2.01)	0.61 (0.18–2.14)	0.24 (0.07–0.85) *	0.38 (0.08–1.67)
Sensitivity to chemicals (QEESI)							
	Low	Ref.					
	High		1.04 (0.46–2.35)	0.72 (0.27–1.87)	1.68 (0.73–3.89)	1.06 (0.48–2.38)	0.98 (0.42–2.27)
Physical condition							
	good	Ref.					
	not good		0.32 (0.07–1.44)	0.33 (0.05–2.17)	0.63 (0.15–2.75)	1.16 (0.30–4.41)	0.72 (0.18–2.84)
Medical history of allergy							
	no	Ref.					
	yes		0.72 (0.31–1.64)	0.39 (0.15–1.04)	0.86 (0.38–1.94)	0.89 (0.39–2.07)	1.00 (0.42–2.35)
Current smoking status							
	no	Ref.					
	yes		0.60 (0.21–1.74)	1.64 (0.47–5.73)	0.74 (0.25–2.21)	0.91 (0.33–2.55)	0.42 (0.13–1.32)
Occurrence of BRSs							
	no	Ref.					
	yes		0.45 (0.10–2.01)	0.06 (0.01–0.37) *	0.15 (0.02–1.28)	0.38 (0.10–1.43)	0.25 (0.03–2.10)
FeNO concentrations							
	normal	Ref.					
	high		0.74 (0.30–1.84)	0.70 (0.25–1.98)	1.03 (0.41–2.60)	0.38 (0.16–0.92) *	0.78 (0.30–2.04)
Salivary amylase assay							
	normal	Ref.					
	high		1.63 (0.52–5.11)	1.41 (0.33–6.01)	1.75 (0.57–5.33)	1.87 (0.56–6.23)	2.53 (0.86–7.38)
Laboratory House							
	LH-A	Ref.					
	LH-B		6.59 (2.95–14.74) *	8.45 (3.05–23.41) *	1.05 (0.47–2.36)	2.57 (1.15–5.73) *	2.86 (1.24–6.61) *

* *p* < 0.05. ** Invalid due to complete separation.

**Table 5:** Results of logistic regression on the associations between objective evaluation and personal and environmental factors.

Factors		Objective	
		(α/β) Wave Rate of Change	
		Odds Ratio (95% CI)	
Gender			
	Male	Ref.	
	Female		0.54 (0.20–1.48)
Age			
	20–29	Ref.	
	30–39		0.94 (0.31–2.89)
	40–49		0.39 (0.09–1.60)
	≥50		1.09 (0.29–4.11)
Sensitivity to chemicals (QEESI)			
	Low	Ref.	
	High		0.71 (0.29–1.73)
Physical condition			
	good	Ref.	
	not good		5.59 (0.60–51.85)
Medical history of allergy			
	no	Ref.	
	yes		1.74 (0.70–4.32)
Current smoking status			
	no	Ref.	
	yes		1.46 (0.50–4.33)
Occurrence of BRSs			
	no	Ref.	
	yes		0.78 (0.15–4.16)
FeNO concentrations			
	normal	Ref.	
	high		1.15 (0.43–3.06)
Salivary amylase assay			
	normal	Ref.	
	high		0.59 (0.17–2.13)
Laboratory house			
	LH-A	Ref.	
	LH-B		3.03 (1.23–7.50) *

* *p* < 0.05. Ref.: Reference

## Data Availability

Not applicable.

## Supplementary Materials

The following are available online at https://www.mdpi.com/1660-4601/18/19/10246/s1, Figure S1: The frequency distribution of the rate change of increase/decrease in α/β value, Figure S2: The relationship of the frequency distribution of the rate of change increase/decrease in alfa/beta values between LH-A and B.
