# Supplementary material for: Assessment of Personal Relaxation in Indoor-Air Environments: Study in Real Full-Scale Laboratory Houses
Source: Int J Environ Res Public Health. 2021 Sep 29;18(19):10246. doi: 10.3390/ijerph181910246 (PMC8549697; doi:10.3390/ijerph181910246)
Supplement: Supplementary file 1 [file ijerph-18-10246-s001.zip › ijerph-1375707-supplementary.pdf]

Supplementary Material

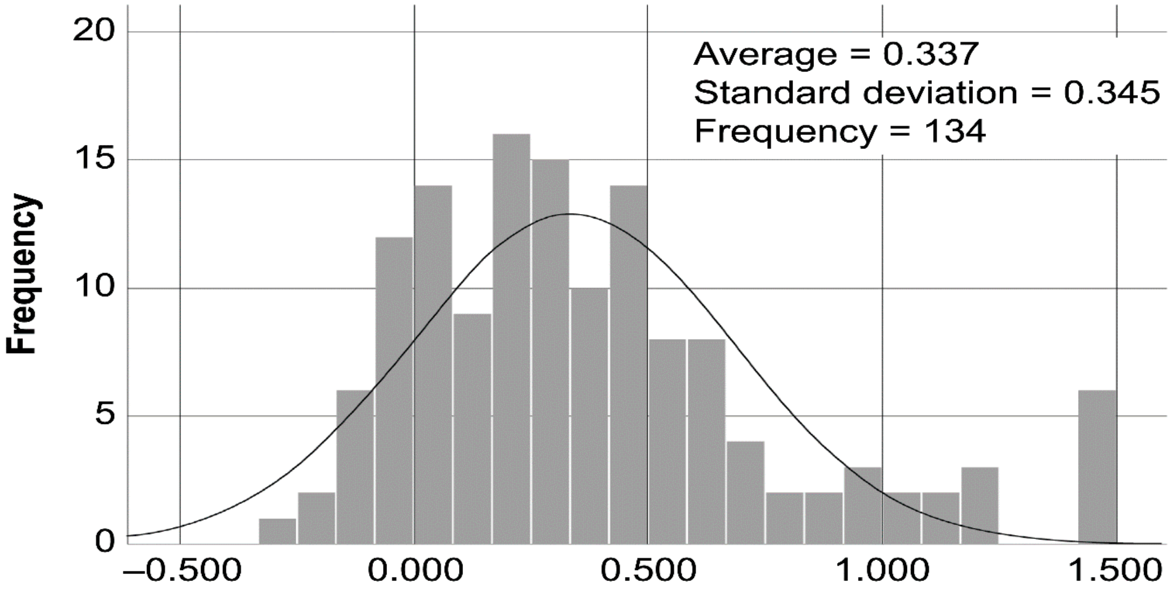

Figure S1. The frequency distribution of the rate change of increase/decrease in α/β value.

The rate of increase or decrease in α/β indicates the ratio of the α/βvalues changed in the task of rest relative to the calculation and memorization tasks and the rate of increase was 84.3% overall. The result of the Shapiro–Wilk test showed a non-normal distribution with a P value of less than 0.05.

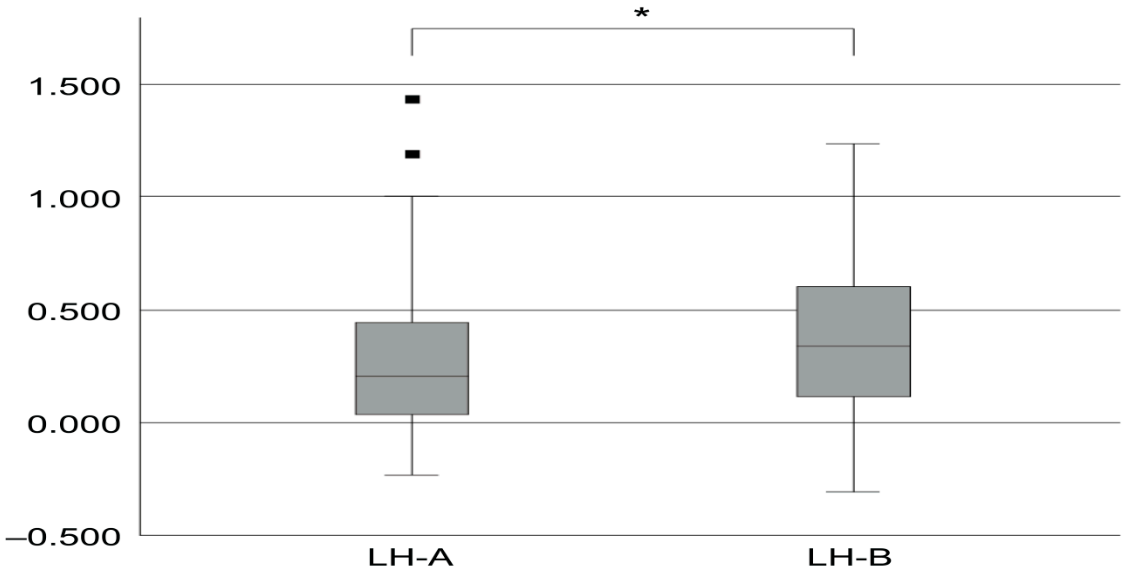

* Mann–Whitney U test, significant at $p < 0.05$

Figure S2. The relationship of the frequency distribution of the rate of change increase/decrease in alfa/beta values between LH-A and B.
